# Ancient schwannoma—a rare cause of mesenteric root expansion

**DOI:** 10.1093/jscr/rjaf486

**Published:** 2025-07-17

**Authors:** Tomas Kriegler, Ondrej Maly, Vladimir Ninger, Miroslav Podhola, Tomas Dusek

**Affiliations:** Department of Surgery, Hospital in Chrudim, Hospital of Pardubice Region, Vaclavska 570, 53727 Chrudim, Czech Republic; Department of Military Surgery, Military Faculty of Medicine in Hradec Kralove, University of Defence in Brno, Trebesska 1575, 50002 New Hradec Kralove, Czech Republic; Department of Military Surgery, Military Faculty of Medicine in Hradec Kralove, University of Defence in Brno, Trebesska 1575, 50002 New Hradec Kralove, Czech Republic; Department of Surgery, University Hospital Hradec, Kralove Sokolska 581, 50005 New Hradec Kralove, Czech Republic; Department of Surgery, Hospital in Chrudim, Hospital of Pardubice Region, Vaclavska 570, 53727 Chrudim, Czech Republic; Fingerland Institute of Pathology, University Hospital Hradec Kralove, Sokolska 581, 50005 New Hradec Kralove, Czech Republic; Department of Military Surgery, Military Faculty of Medicine in Hradec Kralove, University of Defence in Brno, Trebesska 1575, 50002 New Hradec Kralove, Czech Republic; Department of Surgery, Hospital in Pardubice, Hospital of Pardubice Region, Kyjevska 44, 53003 Pardubice, Czech Republic

**Keywords:** schwannoma, expansion of the mesentery, abdominal pain, fatigue, gastrointestinal tract

## Abstract

Ancient schwannoma is a rare cause of expansion of the mesenteric root. It grows from nerve fibers in the mesentery and manifests itself with nonspecific symtomps. The authors present the case of a 53-year-old patient who was examined for 2 months of fatigue and occasional abdominal cramps. Ultrasound examination of the abdomen showed resistance in the left mesogastric area. Blood count and biochemical blood tests were unremarkable. Magnetic resonance imaging did not show activation of lymph nodes during resistance and metastatic spread in the abdominal cavity. The expansion was diagnosed as bening ancient schwannoma by computed tomography biopsy performed before surgery. The patient underwent elective laparoscopic surgery and the tumor was completely removed. Subsequently, the diagnosis was histologically verified postoperatively. Adjuvant therapy and further dispensary for the patient was not indicated due to the benign nature of the tumor. Complete surgical removal of the tumor remains the best therapy.

## Introduction

Schwannomas represent a large group of tumors that grow from Schwann cells of the peripheral nervous system or spinal cord roots. Almost half of schwannomas occur in the head and neck region. A typical and at the same time the most common example of intracranial schwannoma is vestibular schwannoma arising from Schwann cells of the vestibulocochlear nerve [1]. Extracranially and outside of the spinal roots, schwannomas occur significantly more rarely in the area of peripheral nerve fibers of the gastrointestinal tract (GIT) [2]. These are predominantly tumors of a histologically well-differentiated and benign nature [3]. In the GIT area, schwannomas represent 2%–6% of mesenchymal tumors, 70% of them occur in the stomach area, their incidence in the colon and rectum is reported to be ⁓3%, and they occur even more rarely in the area of the small intestine and esophagus [4–6]. Most GIT schwannomas are diagnosed as an incidental finding. Up to 2% of all schwannomas have malignant potential and thus the ability to form metastases [7]. The patient provided written informed consent for this record to be published.

## Case report

**Figure 1:**
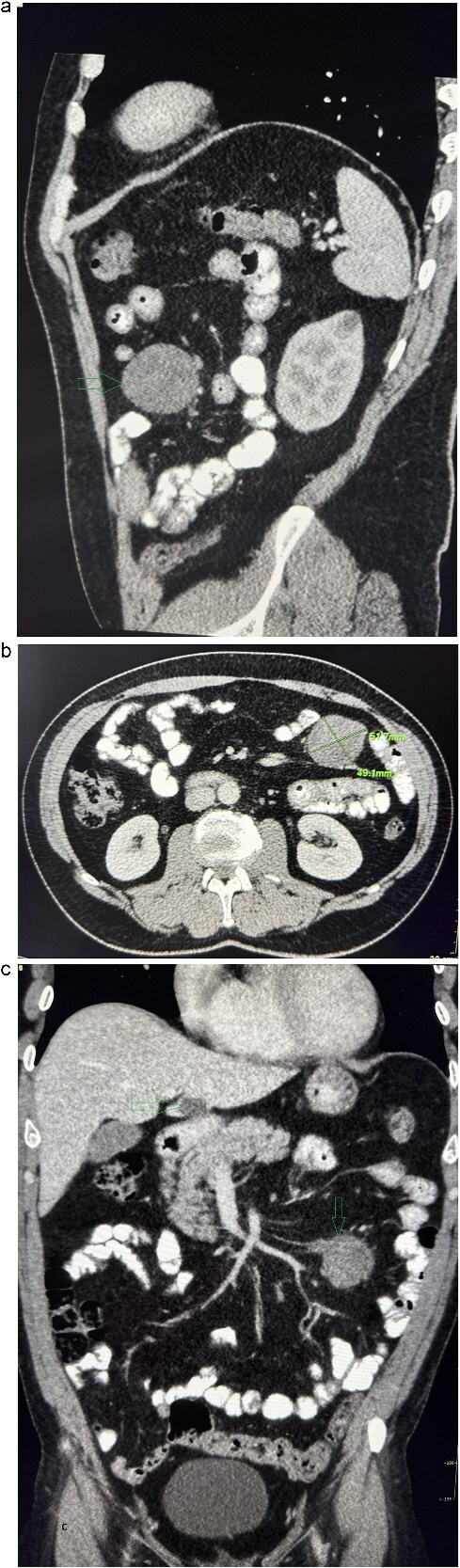
Contrast-enhanced abdominal CT. (a) The sagittal section presents a tumor mass at the root of the mesentery in the left mesogastrium. (b) The axial section presents a tumor mass measuring 52×49 mm in diameter. (c) The coronal section presents a tumor mass at the root of the mesentery in the left mesogastrium and suspected metastatic liver lesion.

**Figure 2:**
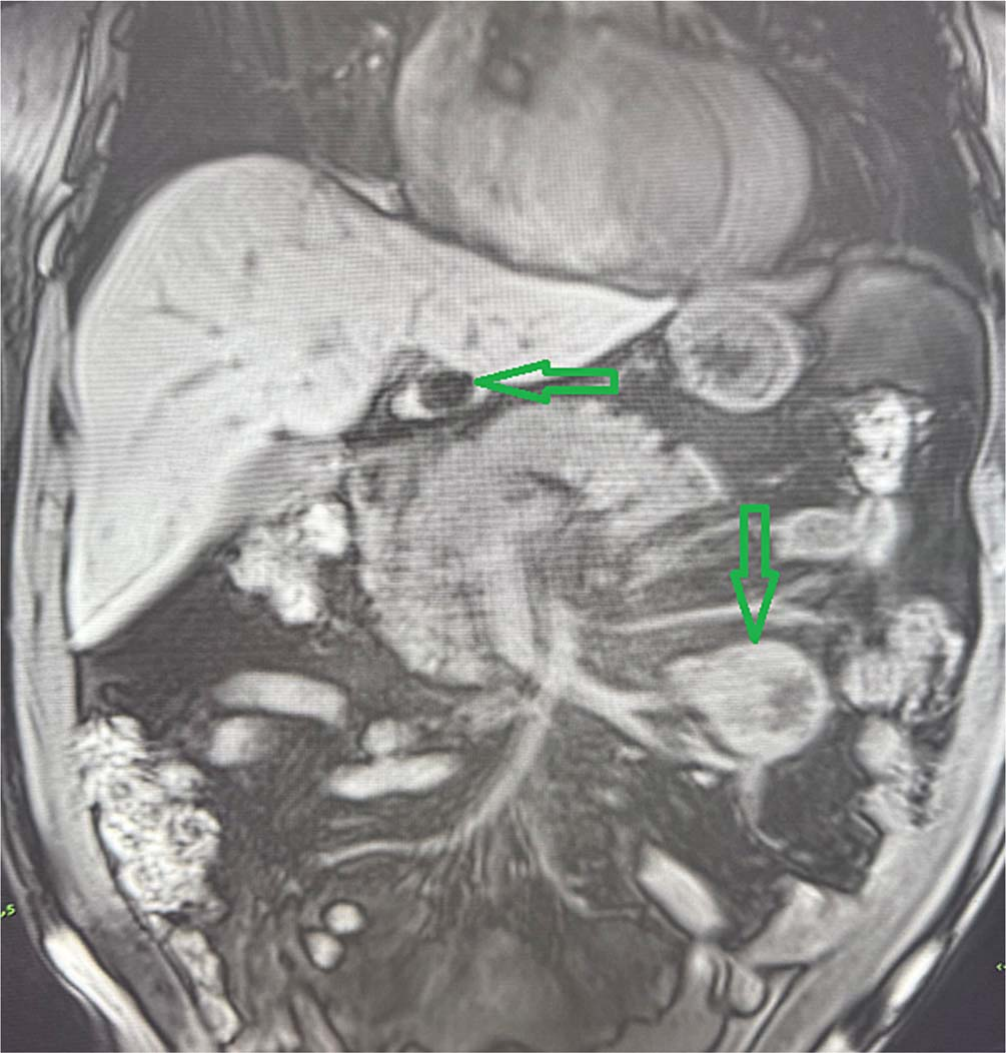
Abdominal MRI, T1 sequency. The coronal section presents a tumor mass at the root of the mesentery in the left mesogatrium and the liver cyst.

**Figure 3:**
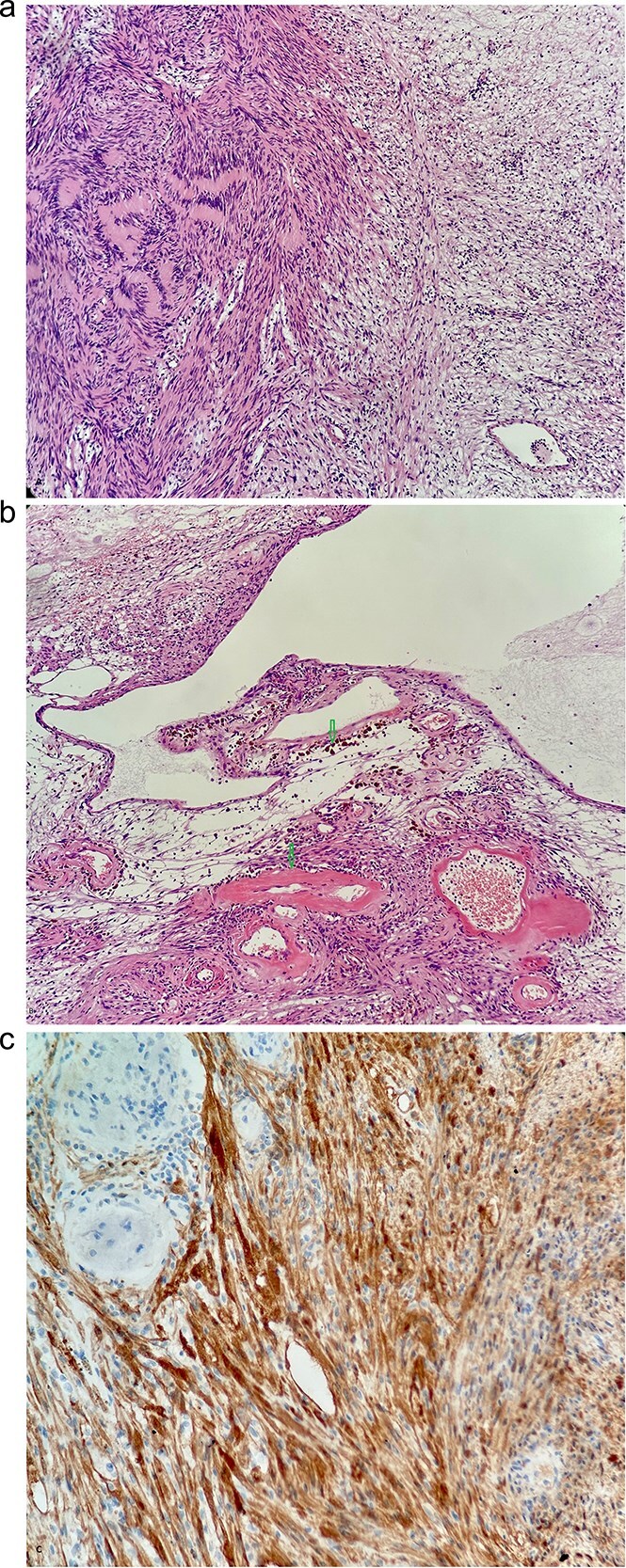
(a) Typical biphasic appearance of schwannoma. The left half of the image shows a compact area with a rhythmic arrangement of nuclei and Verocay bodies (Antoni a). On the right, a sparse hypocellular area is visible (Antoni B)—HeEo ×100. (b) Ancient changes in schwannoma, where pseudocystic transformation with siderofages is evident as a sign of older hemorrhage and hyalinized tumor vessels—HeEo ×100. (c) Detailed strong diffuse positivity (brown cells) of tumor cells in immunohistochemical reaction with antibody against the S100 protein ×200.

A 53-year-old obese polymorbid patient was initially examined by his general practitioner for 2 months of fatigue. Routine blood tests, physical examination, and X-rays of the lungs were unremarkable. Abdominal ultrasound examination diagnosed resistance in the left mesogastrium and no other pathology was found. The patient was referred to the surgical department for further examination. Abdominal examination was supplemented with computed tomography (CT), which diagnosed a circumscribed hypodense mass of 52 mm at the root of the mesentery in the left mesogastrium and suspected solitary metastatic liver disease (Fig. 1). Massive lymphadenopathy was considered in the differential diagnosis of mesenteric root resistance. Magnetic resonance imaging (MRI) was also indicated to verify the CT findings. MRI examination of the abdomen showed a tumor resistance of 58 × 45 × 45 mm in the root of the mesentery in the left mesogastrium and metastatic liver damage, including lymphadenopathy in the mesentery, was also excluded. The liver mass was diagnosed as a cyst (Fig. 2). According to MRI examination, a possible gastrointestinal stromal tumor, neurogenic tumor, or solitary fibrous tumor was considered as part of the differential diagnosis of mesenteric root resistance. The examination of oncological markers was negative. To investigate the etiology of resistance in the root of the mesentery, a biopsy of resistance was performed under CT control. The biopsy examination showed an ancient schwannoma with positive protein S100 and SOX10 on immunohistochemical examination. Based on the decision of the interdisciplinary indication seminar, the patient was indicated for elective surgery, laparoscopically assisted ileum resection with minilaparotomy in the left mesogastrium. The continuity of the ileum was then restored with a terminoterminal anastomosis sewn by hand seromuscularly in one row with a continuous monofilament suture. The operation went without complications and the patient was discharged on the fourth postoperative day for outpatient care with a fully restored intestinal passage. The tumor-resected ileum was sent for histological examination, which confirmed a schwannoma of the mesentery, which was completely removed (Fig. 3). The surgical wounds were mainly healed. No further dispensary for the patient was indicated.

## Discussion

Schwannomas are tumors that arise from excessive proliferation of Schwann cells, which form the myelin sheath of the axons of nerve fibers. Due to their histological origin and the occurrence of Schwann cells in the human body, these tumors can occur anywhere from the peripheral nervous system to the central nervous system, however, they are rarely described in the GIT region [8]. Schwannomas of the GIT are mostly benign origin, but under certain conditions they also have malignant potential, e.g. in some genetic diseases such as neurofibromatosis (type 1 + 2), when the tumor suppressor genes NF1 and NF2 are mutated. These mutations lead to increased mitotic activity of Schwann cells and subsequently to the formation of malignant schwannomas of the GIT [9]. GIT schwannomas can reach different sizes, tumors ˂1 cm are described, but 28 cm tumors can also appear. Schwannomas most often occur solitary, only rarely occur in multiple localization. Their incidence is currently increasing due to the availability of modern diagnostic immunohistochemical methods. The expression of the S100 protein is typical for nerve cell tumors, which show up to 98% specificity, and therefore the S100 protein is considered a specific diagnostic marker. Additional diagnostic markers and markers used in differential diagnosis—CD34 and vimentin give a positive reaction, while markers—neuron-specific enolase, CD117, desmin, and smooth muscle antigen are always negative [10]. The clinical manifestations of GIT schwannomas depend on their location. If they occur in the stomach, they can lead to upper-type dyspepsia. If a tumor occurs in the area of the small or large intestine, it can manifest itself as melena, intestinal obstruction, and can also be a guiding point for intussusception [11]. As part of the differential diagnosis, it is necessary to first exclude GIT cancer, then gastrointestinal stromal tumors, where the malignant potential is reported to be up to 30%, then GIT lymphoma and GIT smooth muscle tumor [12]. Treatment of GIT schwannomas is surgical in most cases, either by classic open surgery or laparoscopically. Endoscopic resection is also possible for tumors up to 3 cm, but its limit is the degree of depth of tumor invasion. When the tumor grows under the mucosa, endoscopic resection represents an increased risk of bleeding, perforation at the site of the procedure, or possible fistulation [13]. The laparoscopic procedure represents a lower burden for the patient and the possibility of faster recovery than traditional open surgery. It can also be combined with an endoscopic procedure. The prognosis after complete resection is excellent. In the case of malignant schwannomas, surgical resection is always indicated, including regional lymphadenectomy, which is not performed as standard, and therapy is supplemented with radiation therapy, chemotherapy, or biological treatment after consultation with the oncologist [14].
